# Response: Commentary: Blood Flow Restriction Exercise: Considerations of Methodology, Application, and Safety

**DOI:** 10.3389/fphys.2021.665568

**Published:** 2021-03-29

**Authors:** Tim Kambic, Borut Jug, Mitja Lainscak

**Affiliations:** ^1^Cardiac Rehabilitation Unit, Department of Research and Education, General Hospital Murska Sobota, Murska Sobota, Slovenia; ^2^Faculty of Medicine, University of Ljubljana, Ljubljana, Slovenia; ^3^Division of Internal Medicine, Department of Vascular Diseases, University Medical Centre Ljubljana, Ljubljana, Slovenia; ^4^Division of Cardiology, General Hospital Murska Sobota, Murska Sobota, Slovenia; ^5^Faculty of Natural Sciences and Mathematics, University of Maribor, Maribor, Slovenia

**Keywords:** blood flow restriction, resistance training, exercise pressor reflex, coronary artery disease, cardiac rehabilitation

We read with great interest the recent comprehensive guidelines for the implementation of blood flow-restricted resistance exercise (BFR-RE) into sports and clinical practice. The authors provided an extensive description of the mechanism and training application and addressed many important safety considerations (Patterson et al., 2019). With the potential of BFR exercise expanded to clinical settings [such as in orthopedic (Hughes et al., 2017) and cardiovascular patients (Madarame et al., 2013; Tanaka and Takarada, 2018; Kambič et al., 2019)], many previous reviews have raised safety concerns (Spranger et al., 2015; Oliveira et al., 2019). These are due to potential peripheral ischemia-induced hyperactivity of III and IV nerve afferents that could evoke muscle metabo- and/or mechanoreflex (e.g., the exercise pressor reflex), primarily in cardiovascular patients (Piepoli et al., 2008; Angius and Crisafulli, 2020). Since the guidelines focused mainly on the effects of BFR-RE on cardiovascular response and blood coagulation (Patterson et al., 2019), the recent commentary in the journal also highlighted this important safety issue (Spranger, 2020).

In the commentary, the potential role of exercise pressor reflex during BFR-RE was linked with a higher increase in blood pressure during low-load BFR-RE compared with low-load RE without BFR (Spranger, 2020), as demonstrated in previous studies enrolling healthy adults (Downs et al., 2014; Hori et al., 2020) and older women (Scott et al., 2018). In contrast, one study, not included in the recent commentary (Spranger, 2020), has demonstrated lower blood pressure during low-load BFR-RE [30% of one-repetition maximum (1-RM), 4 sets of 15 repetitions per set, with 60 s of rest between sets] compared with low-load and high-load RE to failure (Libardi et al., 2017). This indicates that time under BFR is likely a major contributor to more pronounced exercise pressor reflex observed when sets of more than 15 repetitions (Scott et al., 2018; Hori et al., 2020) or sets to volitional fatigue are performed (Downs et al., 2014). Therefore, we agree with the author that future BFR-RE training implementations in cardiovascular rehabilitation settings should take into consideration the duration of time under BFR (e.g., duration of the exercise), applied cuff pressure to the limb, and width of the cuff (Loenneke et al., 2013), as the main mediators of the magnitude of exercise pressor response (Oliveira et al., 2019).

The implementation of BFR-RE in cardiovascular patients (e.g., coronary artery disease, heart failure, and peripheral artery disease) was addressed only in two hemodynamic studies (Pinto and Polito, 2016; Kambič et al., 2020). Both were included in the commentary (Spranger, 2020), yet we argue that several key findings of our study about BFR-RE safety (e.g., hemodynamic response during exercise) were not discussed thoroughly. Importantly, our study also measured hemodynamic response during low-load BFR-RE at 30 and 40% of 1-RM (Kambič et al., 2020), in addition to the already mentioned hemodynamic adaptations after BFR resistance training (RT) (Kambič et al., 2019). Prior to BFR-RT intervention, we measured heart rate and blood pressure response to three sets of 8, 10, and 12 repetitions at the intensity of 30% of 1-RM, a lifting cadence of 1 s of concentric contraction and 2 s of eccentric contraction, and with 45 s of rest between sets (Kambič et al., 2020). Heart rate (HR), systolic blood pressure (SBP), and diastolic blood pressure (DBP) increased significantly in the first set (HR: +10 bpm, SBP: +12 mmHg, DBP: +3 mmHg), second set (HR: +14 bpm, SBP: +22 mmHg, DBP: +10 mmHg), and third set (HR: +18 bpm, SBP: +13 mmHg, DBP: +3 mmHg) compared with baseline levels. Furthermore, HR, SBP, and DBP increased significantly from the second set to the third set, while BP was significantly lower after the cuff pressure was released after the third set compared with the second set. All hemodynamic parameters returned to baseline values after the end of BFR-RE. After the completion of 8 weeks of BFR-RT intervention, we re-evaluated the hemodynamic response to BFR-RE at the intensity of 40% of 1-RM. With the exception of lower diastolic pressure in the third set compared with the first of the BFR-RE, leading to a significant set × intensity interaction (*p* = 0.027), we observed a similar increase in HR and SBP as during the baseline measurement at 30% of 1-RM, with no significant set × intensity interaction. In addition, BFR-RE did not induce any changes in circulating levels of hemostatic markers (D-dimer and fibrinogen) and N-terminal prohormone B-type natriuretic hormone following acute BFR-RE and BFR-RT (Kambič et al., 2020), which is in line with the only study available in coronary artery disease patients (Madarame et al., 2013).

Despite our novel findings on the safety and efficacy of BFR-RT on muscle strength and vascular function, there remain many methodological limitations and unanswered questions. These should be addressed in future trials before BFR-RE can be routinely included in cardiac rehabilitation. Ideally, future trials should use indirect beat-by-beat methods (photoplethysmography or impendance cardiography) (Downs et al., 2014; Scott et al., 2018) or direct measurements of hemodynamic response using an arterial and venous catheter on the exercising limb (Franz et al., 2020); as these methods are not used routinely, a correlation study with usual hemodynamic monitors (automated BP monitor) should be considered. Future trials should also be designed to study the hemodynamic effects of high-load RE (>70% 1-RM) and low-load RE with and without BFR (<40% 1-RM). In addition, special consideration should be given to the selection of narrow cuffs (Loenneke et al., 2013) and the reduction of time under BFR, with manipulation of the number of sets (<3–4 sets) and repetitions (<15 repetitions per set) (Madarame et al., 2013; Kambič et al., 2019), and lifting cadence (1 s:1 s of concentric and eccentric contraction) (Lamotte et al., 2010) to minimize the (potential) activation of exercise pressor reflex in cardiovascular disease patients.

## Author Contributions

TK: writing of the manuscript draft and responsible for the final content. BJ and ML: writing of the manuscript draft. All authors read, critically reviewed, and approved the final version of the manuscript.

## Conflict of Interest

The authors declare that the research was conducted in the absence of any commercial or financial relationships that could be construed as a potential conflict of interest.
